# Cladribine Exposure Results in a Sustained Modulation of the Cytokine Response in Human Peripheral Blood Mononuclear Cells

**DOI:** 10.1371/journal.pone.0129182

**Published:** 2015-06-18

**Authors:** Melanie Korsen, Sara Bragado Alonso, Lizzy Peix, Barbara M. Bröker, Alexander Dressel

**Affiliations:** 1 Section of Neuroimmunology, Department of Neurology, University Medicine Greifswald, Greifswald, Germany; 2 Center for Regenerative Therapies, DFG-Research Center and Cluster of Excellence Dresden, Dresden, Germany; 3 Department of Inflammation and Tissue Repair, Centre for Respiratory Research, University College London, London, United Kingdom; 4 Department of Immunology, University Medicine Greifswald, Greifswald, Germany; Fraunhofer Institute for Cell Therapy and Immunology, GERMANY

## Abstract

**Background and Objectives:**

Cladribine is a cytotoxic drug which ameliorates the clinical course of relapsing-remitting multiple sclerosis. In addition to cytotoxicity, the mode of action may include immunomodulatory mechanisms. This *in vitro* study was designed to investigate cladribine’s effects on cell function after the removal of cladribine to distinguish cytotoxic versus immunomodulatory effects.

**Methods:**

Cells were incubated in the absence or presence of cladribine (1×10^-8^ M to 1×10^-5^ M) for 72 h. Cladribine was removed from the cell culture and surviving peripheral blood mononuclear cells were cultured up to 58 days to determine the immunomodulatory effects of cladribine on cell function (e.g., proliferation and cytokine release).

**Results:**

In the long-term, brief cladribine exposure did not impair the proliferation of surviving peripheral blood mononuclear cells. However, it induced an anti-inflammatory shift in the cytokine milieu with significantly enhanced release of IL-4 (Days 9 and 44, p<0.01; Day 58, p<0.05) and IL-5 (Day 9, p<0.01), resulting in an increased IL-4/INF-gamma ratio (Days 9 and 44, p<0.01; Day 58, p<0.05). Additionally, a trend towards an increased IL-10 production was observed. No changes were found in the production of IFN-gamma, TNF-alpha, IL-6, IL-8, IL-17A, IL-23 or NGF-beta.

**Conclusions:**

*In vitro* cladribine exposure induces a sustained anti-inflammatory shift in the cytokine profile of surviving peripheral blood mononuclear cells. This immunomodulatory action might contribute to cladribine’s beneficial effects in the treatment of multiple sclerosis.

## Introduction

Multiple sclerosis (MS) is a chronic inflammatory disease of the central nervous system which affects more than two million patients worldwide and is the most frequent cause of non-traumatic disabilities in young adults [1,2]. Due to the fact, that there is still no curative treatment for MS available pharmaceuticals aim for a reduction in relapse rate, disability progression, neurological degeneration and a symptomatic treatment of disease symptoms [3].

Clinical phase III trials have demonstrated a beneficial effect on the clinical course of relapsing remitting MS for at least 10 compounds. The mechanism of action of these drugs is considered either immunomodulatory (e.g., interferon-beta, glatirameracetate) or immunosuppressive (e.g., mitoxantrone, cyclophosphamide and teriflunomide) [4–6].

Cladribine, a synthetic purine nucleotide analogue of desoxyadenosine is one of the medications that has shown clinical efficacy in a phaseIII clinical trial in RRMS [6,7]. The compound is cytotoxic to proliferating and non-proliferating lymphocytes, as well as to monocytes and monocyte-derived dendritic cells [8–11]. It is currently used as part of therapy against malignant haematological diseases (e.g., hairy cell leukaemia, chronic lymphatic leukaemia, chronic and acute myeloid leukaemia and non-Hodgkin lymphomas) [12].

In its phase III clinical trial in RRMS patients, oral cladribine was administered in two or four courses of treatment within the first 48 weeks, followed by two additional courses during Weeks 48 and 52. This brief treatment resulted in a significant reduction of the relapse rate, magnetic resonance imaging (MRI) activity and disability progression in RRMS patients [7,13]. Despite meeting its primary outcome parameter during the phaseIII clinical trial, the Food and Drug Administration and the European Medicine Agency refused marketing authorisation for cladribine because of its benefit risk profile [14]. Compared with placebo, lymphopenia and *Herpes zoster* virus infections were more likely to occur in patients receiving cladribine. Furthermore, a slightly higher risk of developing secondary malignancies had not been ruled out [7,15].

The beneficial effects observed in RRMS patients persisted for many months after the cessation of cladribine exposure, which was limited to two or four courses within the first 48 weeks followed by two courses during Weeks 48 and 52 [7,13]. Therefore, we hypothesized that in addition to its direct cytotoxic effects the compound’s mode of action may also encompass immunomodulatory mechanisms. Such effects might include alterations in cytokine patterns of cells that survived the initial cladribine treatment. Indeed, cyclophosphamide and mitoxantrone, two other immunosuppressive drugs, are well known to have additional immunomodulatory effects [16–18].

For cladribine several studies demonstrated reductions in the release of pro- and anti-inflammatory cytokines, chemokines, and adhesion molecules, as well as a decrease in the cellular migratory capacity of treated cells [19–21]. However, these experiments were performed in the presence of cladribine; therefore, toxic effects rather than modulation of cell function could account for the observed changes. The current *in vitro* study was designed to explore whether cladribine exerts long-term immunomodulatory effects in addition to immediate cytotoxicity.

## Material and Methods

### Cell isolation

Peripheral blood mononuclear cells (PBMCs) were isolated from buffy coats of male and female blood donors (Transfusion Medicine, University Medicine Greifswald, Germany) using standard Ficoll gradient centrifugation. CD4^+^ cells and CD8^+^ cells were positively selected using Dynal CD4 Positive Isolation Kit and Dynal CD8 Positive Isolation Kit (Invitrogen GmbH, Karlsruhe, Germany) according to the manufacturer’s instructions. All cells were freshly isolated directly before their use. No cryopreserved cells were used in any experiment.

### Cell culture

Cells were cultured in RPMI-1640 growth medium (Sigma-Aldrich Chemie GmbH, Steinheim, Germany) supplemented with 1% glutamine, 1% penicillin, 1% streptomycin and 10% human AB serum (Department of Transfusion Medicine, University Medicine Greifswald, Germany). They were stored in an incubator at 37°C and 5% carbon dioxide under a saturated steam atmosphere.

**Fig 1:**
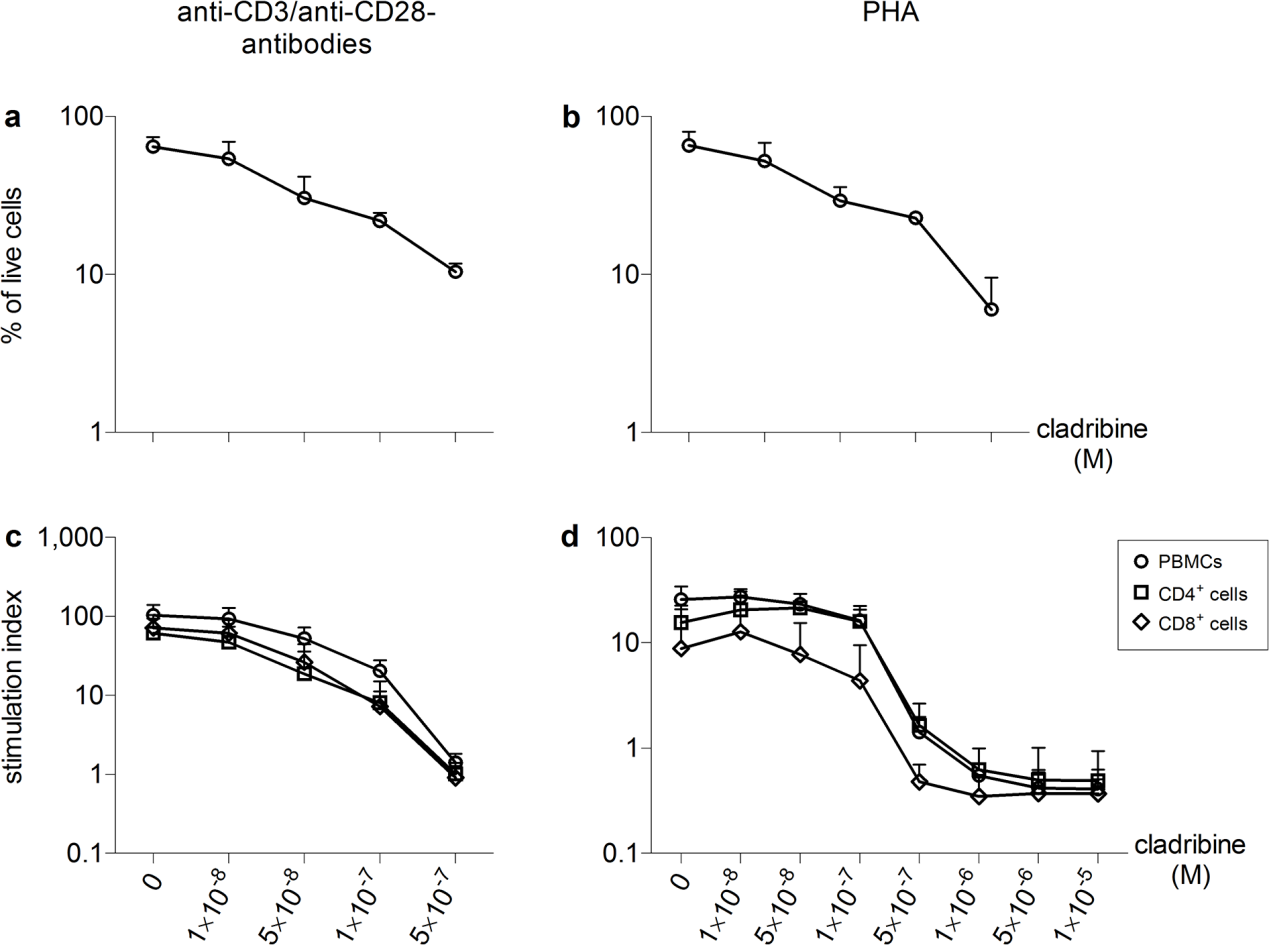
*In vitro* effects of cladribine on cell survival of PBMCs and proliferation of PBMCs, CD4^+^ cells and CD8^+^ cells Human immune cells were stimulated with anti-CD3/anti-CD28 antibodies (a, n = 4; c, n = 6) or PHA (b, n = 4; d, n = 3) in the absence or presence of cladribine (1×10^−8^ M to 1×10^−5^ M) for 72 h. Determination of cell survival in PBMCs (a, b) was performed by standard trypan blue exclusion method. Proliferation was determined separately in PBMC, CD4^+^ cells and CD8^+^ cells (c, d) by the incorporation of tritiated thymidine. Stimulation indices were calculated as the ratios of the counts per minute of stimulated samples to unstimulated and untreated samples. Data are depicted as mean + standard deviation (SD).

Cells were stimulated with either plate-bound anti-CD3 (clone HIT3, 1μg/ml, BD Biosciences Cat# 555336, RRID: AB_395742) and plate-bound anti-CD28 (clone CD28.2, 1μg/ml, BD Biosciences Cat# 555725, RRID: AB_396068) antibodies (BD Labware, Franklin Lakes, NJ, USA) or phytohaemagglutinin (PHA, 1μg/ml) or remained unstimulated. In all experiments PBMCs, CD4^+^ cells and CD8^+^ cells were incubated in the presence (1×10^-8^M to 1×10^-5^M) or absence of cladribine [Leustatin (1mg/ml cladribine, sodium chloride, sodium phosphate dibasic heptahydrate), Janssen-Cilag GmbH, Neuss, Germany or Litak (2mg/ml cladribine, sodium chloridhydroxid, hydrochloric acid) Lipomed GmbH, Weil am Rhein, Germany]. The latter was only used for the survival experiments represented in Fig 1A and 1B. Cladribine was provided in dissolved form and was further diluted to the appropriate concentration with RPMI-1640 growth medium (Sigma-Aldrich Chemie GmbH, Steinheim, Germany) containing 1% glutamine, 1% penicillin, 1% streptomycin and 10% AB serum. Cladribine was added to the culture medium directly before use. Exposed cells were treated with cladribine for 72 h. In all experiments, PBMCs, CD4^+^ cells and CD8^+^ cells were seeded with a cell density of 200,000 cells per well.

In short-term experiments, cell survival was determined by counting of live cells using the standard trypan blue exclusion method in single use C-Chip Neubauer improved counting chambers (NanoEnTec Newton, MA, USA). A total number of four independent experiments were performed. Proliferation was determined by assessing the incorporation of tritiated thymidine. After 72 h, tritiated thymidine was added to the culture and incorporation was quantified using a Phosphorimager (Molecular Dynamics, Sunnyvale, USA). Proliferation was evaluated as the stimulation index, which was calculated as the ratio of counts per minute of sample with stimulation to counts per minute of untreated sample without stimulation. A total number of six (stimulation with anti-CD3/anti-CD28 antibodies) and three (PHA stimulation) independent experiments were performed with a minimum of three technical replicates per independent experiment. Apoptosis was assessed using Annexin V and propidium iodide staining after 12, 24, 48 and 72 h (FITC Annexin V Apoptosis Detection Kit, BD Pharmingen, USA) according to the manufacturer’s instructions. Apoptosis was quantified on a FACScan (Becton Dickinson, Franklin Lakes, NJ, USA). Three independent experiments were performed with a minimum of three technical replicates per independent experiment.

For long-term experiments, stimulated PBMCs that were exposed to cladribine (1×10^-8^M to 5×10^-7^M) for the initial 72 h, were washed three times to remove cladribine and transferred into cladribine-free medium to which 50U/ml interleukin (IL)-2 had been added. Cells were cultured for 9 to 58 additional days. At Days 9, 16, 23, 30, 44 and 58, cell numbers were equilibrated and cells were re-stimulated with plate-bound anti-CD3/anti-CD28 antibodies or PHA as described. The re-stimulation was performed in the absence of cladribine and after a 20 h IL-2–free resting phase. Forty-eight hours later, cell proliferation was determined through the incorporation of tritiated thymidine, which was measured using a Microbeta^2^ Plate Counter (PerkinElmer, Waltham, MA, USA). Supernatants were collected for cytokine analysis. A total number of five (stimulation with anti-CD3/anti-CD28 antibodies) and four (PHA stimulation) independent experiments were performed. Number of technical replicates at Days 9, 16, 23, 30, 44 and 58 were adapted to cell availability with a minimum number of three technical replicates per independent experiment.

### Cytokines

Cytokine concentrations were determined using a multiplex assay according to the manufacturer’s instructions (FlowCytomix Human Th1/Th2 11 Plex Kit, FlowCytomix Human IL17-A, IL-23 and NGF Kit, Bender MedSystems GmbH, Vienna, Austria). A FACScan was used for quantification (Beton Dickinson, Franklin Lakes, NJ, USA). Data were evaluated using the Flow Cytomix Pro 2.2 software (Bender MedSystems GmbH, Vienna, Austria). The IL-4/Interferon-gamma (IFN-γ) ratio was defined as the ratio of IL-4 [pg/ml] to IFN-γ [pg/ml].

### Statistical analysis

This study was designed as an exploratory study, therefore no predefined primary or secondary endpoints were used and statistical significance was not adjusted for multiple testing. Because not all data sets showed Gaussian distributions, the Kruskal-Wallis test and Dunn's Multiple Comparison test were used in all analyses (Prism 5.0 software, GraphPad Software inc., San Diego, CA, USA). P-values <0.05 were considered statistically significant. Data from short-term experiments and proliferation data from long-term experiments are depicted as mean + standard deviation. Cytokine data are presented as box plot diagrams.

### Ethics statement

Blood donations for buffy coats were obtained from healthy donors at the Department of Transfusion Medicine of the University Medicine Greifswald. Written informed consent was obtained from every blood donor on the date of blood donation stating that blood components which will not be used for clinical purposes may be used for clinical investigations and research in an anonymous manner. The Ethics Committee of the University of Greifswald has approved this procedure (BB014/14). Therefore all buffy coats were obtained from individuals that had given written informed consent for the use of their blood cells in a research project but without re-informing the blood donors about the specific study for which their samples were used. The study was conducted according to the principles expressed in the Declaration of Helsinki.

## Results

### Short-term effects of cladribine on cell survival, immune cell proliferation and apoptosis

**Fig 2:**
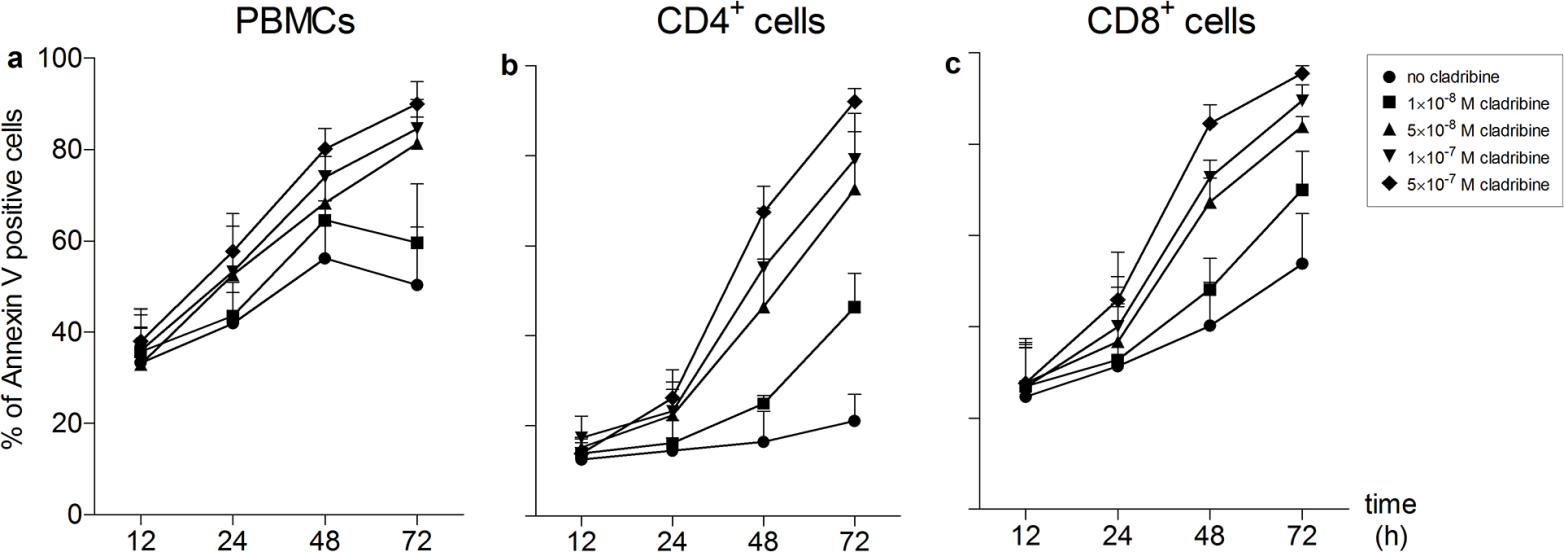
Apoptosis of stimulated PBMCs, CD4^+^ cells and CD8^+^ cells caused by cladribine *in vitro* PBMCs (a, n = 3), CD4^+^ cells (b, n = 3) and CD8^+^ cells (c, n = 3) were stimulated with anti-CD3/anti-CD28 antibodies and were incubated in the absence or presence of cladribine (1×10^−8^ M to 5×10^−7^ M) for 12, 24, 48 and 72 h. Apoptotic cells were defined as Annexin V positive cells. Data are depicted as mean + standard deviation (SD).

Cell survival of PBMCs was determined by microscopic cell counting and trypan blue exclusion of dead cells. At the highest concentration of 5x10^7^ M cladribine, 10% ± 1.3% (mean ± SD) of cells survived after 72 h when stimulated with anti-CD3/anti-CD28 antibodies and 8% ± 4% (mean ± SD) of cells when stimulated with PHA (Fig 1A and 1B). We assessed the effects of increasing concentrations of cladribine on the proliferation of PBMCs, CD4^+^ cells and CD8^+^ cells that were either stimulated with anti-CD3/anti-CD28 antibodies (Fig 1C) or PHA (Fig 1D). When stimulated through their T-cell receptor, 1×10^-7^M cladribine reduced proliferation of PBMCs by 80% and had a slightly stronger effect on T-cell subsets (Fig 1C). PHA-stimulated cells were strongly inhibited in their proliferation starting at a concentration of 5×10^-7^M cladribine (Fig 1D). The decrease in the proliferative response was paralleled by a strong increase in the percentage of Annexin V positive cells, showing that nanomolar concentrations of cladribine are capable of inducing apoptotic cell death (Fig 2, Table 1, Table 2 and Table 3).

**Table 1:** Apoptosis of stimulated PBMCs caused by cladribine *in vitro*.

Condition		Living cells		Early apoptosis		Late apoptosis		Non-apoptotic death	
		Mean in %	SD	Mean in %	SD	Mean in %	SD	Mean in %	SD
PBMCs									
12h	No cladribine	66.7	5.2	22.1	2.0	10.8	3.7	0.4	0.3
	1x10^-8^ M cladribine	64.1	6.6	23.9	3.6	11.5	3.3	0.5	0.3
	5x10^-8^ M cladribine	67.0	5.5	22.3	3.4	10.2	2.4	0.4	0.4
	1x10^-7^ M cladribine	63.5	4.8	24.2	3.1	11.4	2.5	0.8	1.0
	5x10^-7^ M cladribine	61.5	5.4	24.7	4.6	13.0	2.1	0.8	0.9
24h	No cladribine	59.0	6.8	27.5	7.6	13.0	3.5	0.6	0.3
	1x10^-8^ M cladribine	57.3	6.8	28.0	6.0	14.2	4.5	0.4	0.2
	5x10^-8^ M cladribine	48.5	4.2	31.7	9.3	19.4	9.6	0.4	0.3
	1x10^-7^ M cladribine	47.5	8.8	29.5	9.9	22.4	11.4	0.6	0.5
	5x10^-7^ M cladribine	42.9	7.1	30.8	10.0	25.7	13.1	0.5	0.3
48h	No cladribine	42.3	3.9	35.4	13.2	21.9	9.8	0.4	0.5
	1x10^-8^ M cladribine	34.4	3.7	38.8	15.1	26.4	13.0	0.4	0.5
	5x10^-8^ M cladribine	30.2	7.5	32.9	4.6	36.2	11.3	0.7	0.7
	1x10^-7^ M cladribine	25.0	6.0	32.9	10.9	41.5	14.8	0.6	0.7
	5x10^-7^ M cladribine	19.1	5.7	31.3	8.3	49.1	13.0	0.5	0.5
72h	No cladribine	45.6	9.4	27.8	4.8	23.7	8.8	2.9	4.3
	1x10^-8^ M cladribine	37.0	9.3	31.2	1.8	29.3	13.1	2.5	3.6
	5x10^-8^ M cladribine	16.7	4.4	36.9	11.8	45.2	17.9	1.2	1.8
	1x10^-7^ M cladribine	13.6	5.3	35.5	4.6	49.6	11.6	1.2	1.9
	5x10^-7^ M cladribine	8.4	3.9	28.6	2.1	61.8	7.6	1.2	1.8

PBMCs (a, n = 3) were stimulated with anti-CD3/anti-CD28 antibodies and were incubated in the absence or presence of cladribine (1×10^−8^ M to 5×10^−7^ M). Apoptosis was determined after 12, 24, 48 and 72 h by Annexin V and propidium iodide (PI) staining. The table lists the percentages of living cells (Annexin V negative, PI negative cells), cells that are in the state of early apoptosis (Annexin V positive, PI negative cells), cells that are in the state of late apoptosis (Annexin V positive, PI positive cells) and cells that have died a non-apoptotic cell death (Annexin V negative, PI positive cells).

**Table 2:** Apoptosis of stimulated CD4^+^ cells caused by cladribine *in vitro*.

Condition		Living cells		Early apoptosis		Late apoptosis		Non-apoptotic death	
		Mean in %	SD	Mean in %	SD	Mean in %	SD	Mean in %	SD
CD4^+^ cells									
12h	No cladribine	88.4	4.7	4.4	2.9	7.0	1.9	0.3	0.2
	1x10^-8^ M cladribine	87.2	4.7	4.9	3.5	7.7	1.3	0.3	0.2
	5x10^-8^ M cladribine	86.2	3.9	5.3	2.6	8.2	1.3	0.3	0.2
	1x10^-7^ M cladribine	84.2	2.2	5.4	2.5	10.0	0.4	0.4	0.3
	5x10^-7^ M cladribine	86.9	4.5	4.8	2.6	7.9	2.1	0.3	0.3
24h	No cladribine	86.3	3.1	3.7	0.9	9.8	2.3	0.2	0.1
	1x10^-8^ M cladribine	84.5	5.1	4.0	1.3	11.3	3.9	0.2	0.1
	5x10^-8^ M cladribine	78.3	5.7	7.1	3.2	14.4	3.7	0.2	0.1
	1x10^-7^ M cladribine	77.3	6.6	6.8	2.7	15.6	4.6	0.3	0.1
	5x10^-7^ M cladribine	74.5	6.1	8.9	3.5	16.4	4.3	0.2	0.1
48h	No cladribine	82.5	11.4	5.4	5.8	12.0	5.8	0.1	0.0
	1x10^-8^ M cladribine	74.0	5.6	8.2	4.6	17.6	1.0	0.2	0.0
	5x10^-8^ M cladribine	52.7	9.6	12.2	5.7	34.9	5.5	0.2	0.1
	1x10^-7^ M cladribine	44.6	13.2	12.3	5.0	43.0	8.4	0.2	0.1
	5x10^-7^ M cladribine	32.4	7.6	11.7	3.9	55.7	3.9	0.2	0.0
72h	No cladribine	78.6	9.7	5.8	4.5	15.4	5.2	0.2	0.1
	1x10^-8^ M cladribine	53.4	6.7	9.6	3.3	36.7	4.6	0.3	0.1
	5x10^-8^ M cladribine	27.0	12.0	10.2	3.6	62.6	9.8	0.3	0.2
	1x10^-7^ M cladribine	20.4	9.3	9.2	3.7	70.2	9.1	0.3	0.2
	5x10^-7^ M cladribine	7.7	2.7	7.2	3.0	84.9	2.3	0.2	0.2

CD4^+^ cells (a, n = 3) were stimulated with anti-CD3/anti-CD28 antibodies and were incubated in the absence or presence of cladribine (1×10^−8^ M to 5×10^−7^ M). Apoptosis was determined after 12, 24, 48 and 72 h by Annexin V and propidium iodide (PI) staining. The table lists the percentages of living cells (Annexin V negative, PI negative cells), cells that are in the state of early apoptosis (Annexin V positive, PI negative cells), cells that are in the state of late apoptosis (Annexin V positive, PI positive cells) and cells that have died a non-apoptotic cell death (Annexin V negative, PI positive cells).

**Table 3:** Apoptosis of stimulated CD8^+^ cells caused by cladribine *in vitro*.

Condition		Living cells		Early apoptosis		Late apoptosis		Non-apoptotic death	
		Mean in %	SD	Mean in %	SD	Mean in %	SD	Mean in %	SD
CD8^+^ cells									
12h	No cladribine	76.3	14.6	7.6	4.4	15.6	10.1	0.5	0.4
	1x10^-8^ M cladribine	73.9	13.2	9.2	4.5	16.3	8.5	0.6	0.5
	5x10^-8^ M cladribine	73.2	13.4	10.5	5.3	15.9	8.1	0.3	0.3
	1x10^-7^ M cladribine	74.4	12.7	10.4	5.6	14.9	7.3	0.3	0.4
	5x10^-7^ M cladribine	73.7	12.6	11.3	5.4	14.7	7.4	0.4	0.4
24h	No cladribine	70.2	13.8	8.0	3.4	21.7	13.0	0.2	0.1
	1x10^-8^ M cladribine	69.1	13.1	9.8	2.8	20.9	12.8	0.3	0.3
	5x10^-8^ M cladribine	64.8	12.8	11.4	2.5	23.2	12.4	0.5	0.7
	1x10^-7^ M cladribine	61.7	11.3	11.9	5.5	26.1	14.7	0.3	0.3
	5x10^-7^ M cladribine	55.6	11.6	13.8	5.8	29.9	13.9	0.7	0.9
48h	No cladribine	60.9	15.5	8.6	4.1	30.3	11.6	0.2	0.2
	1x10^-8^ M cladribine	52.8	13.9	11.0	4.5	36.1	9.4	0.1	0.1
	5x10^-8^ M cladribine	33.7	11.3	12.8	3.3	53.3	8.3	0.2	0.1
	1x10^-7^ M cladribine	28.0	9.1	12.5	1.3	59.3	7.9	0.2	0.1
	5x10^-7^ M cladribine	16.1	7.6	10.6	1.0	73.1	7.2	0.2	0.1
72h	No cladribine	49.2	14.0	8.6	1.9	41.9	12.2	0.3	0.4
	1x10^-8^ M cladribine	31.3	8.3	10.7	3.1	57.8	10.1	0.3	0.3
	5x10^-8^ M cladribine	17.0	4.6	8.4	2.1	74.3	2.8	0.3	0.3
	1x10^-7^ M cladribine	11.0	3.9	6.8	2.3	82.0	3.2	0.2	0.3
	5x10^-7^ M cladribine	4.7	2.2	5.1	2.0	89.9	1.8	0.3	0.3

CD8^+^ cells (a, n = 3) were stimulated with anti-CD3/anti-CD28 antibodies and were incubated in the absence or presence of cladribine (1×10^−8^ M to 5×10^−7^ M). Apoptosis was determined after 12, 24, 48 and 72 h by Annexin V and propidium iodide (PI) staining. The table lists the percentages of living cells (Annexin V negative, PI negative cells), cells that are in the state of early apoptosis (Annexin V positive, PI negative cells), cells that are in the state of late apoptosis (Annexin V positive, PI positive cells) and cells that have died a non-apoptotic cell death (Annexin V negative, PI positive cells).

### Long-term effects of cladribine on the proliferation of surviving PBMCs

**Fig 3:**
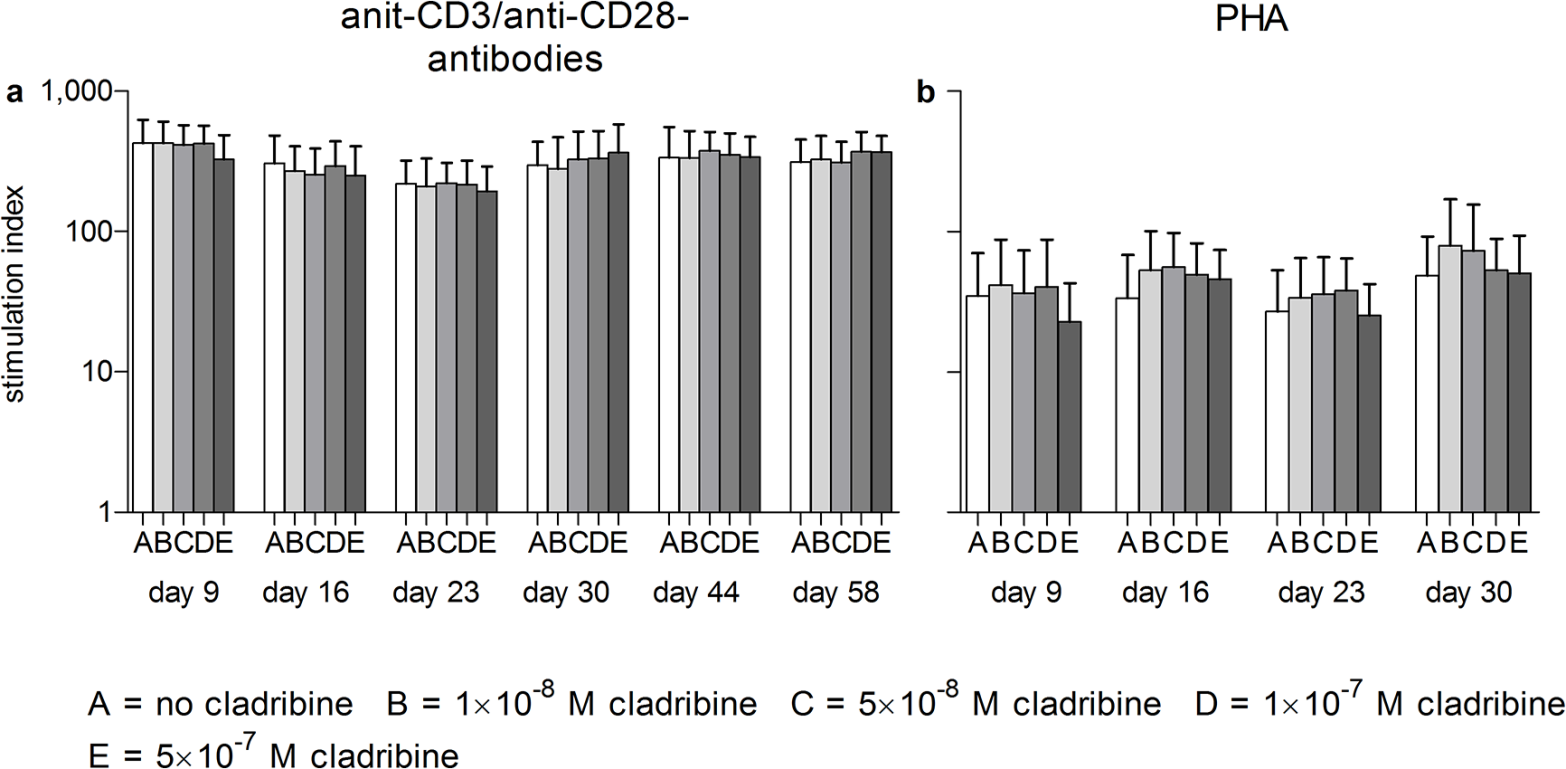
*In vitro* effects of initial cladribine treatment on the proliferation of PBMCs stimulated at Days 9, 16, 23, 30, 44 and 58 after transfer into cladribine-free medium PBMCs were initially incubated in the absence or presence of cladribine (1×10^−8^ M to 5×10^−7^ M) for 72 h. Cells were washed three times and then transferred into cladribine-free medium. Cells were restimulated with anti-CD3/anti-CD28 antibodies (a, n = 5) or PHA (b, n = 4) for 48h at Days 9, 16, 23, 30, 44 and 58; proliferation was determined by the incorporation of tritiated thymidine. Stimulation indices were defined as the ratios of the counts per minute of stimulated samples to unstimulated and untreated samples. Data are depicted as mean + standard deviation (SD).

To evaluate whether *in vitro* treatment with cladribine alters the cytokine profile of surviving cells, proliferation and cytokines were measured after cladribine had been removed from the cell culture medium. Therefore, cladribine was removed from the cell culture after 72 h of incubation, and PBMCs were cultured for up to 58 days in cladribine-free medium supplemented with 50U/ml of the survival factor IL-2. Surviving cells were re-stimulated with anti-CD3/anti-CD28 antibodies or PHA 9 to 58 days after the removal of cladribine. IL-2 was removed from the cell culture 20 h before re-stimulation. Despite the cytotoxic effects observed in short-term treatment (Figs 1 and 2) initial exposure to cladribine did not impair the proliferation of surviving cells (Fig 3).

### Effects of initial cladribine exposure on cytokine release from surviving PBMCs

**Fig 4:**
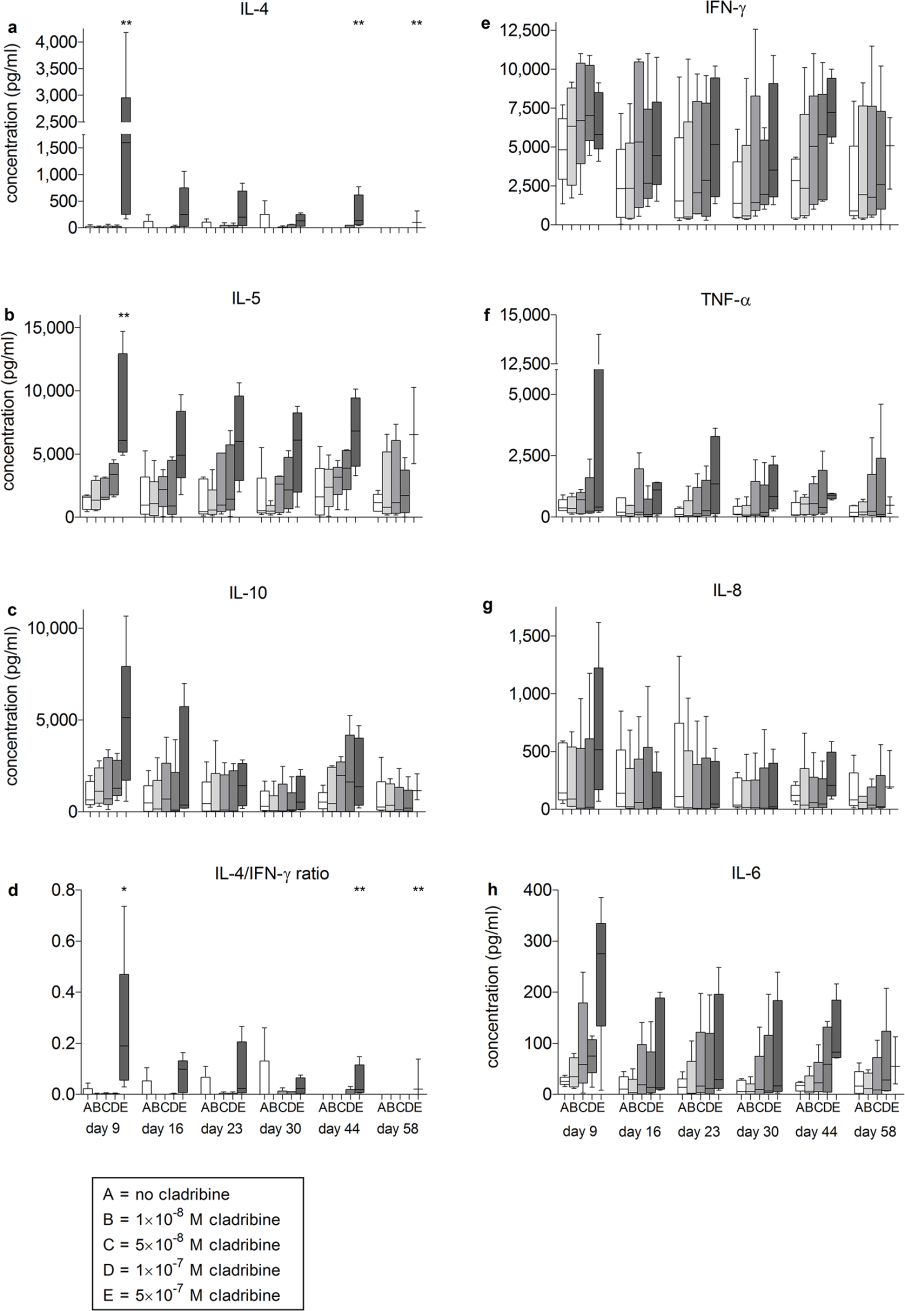
*In vitro* effects of initial cladribine treatment on cytokine secretion of PBMCs restimulated at Days 9, 16, 23, 30, 44 and 58 after transfer into cladribine-free medium. PBMCs from healthy blood donors (n = 5) were incubated in the absence or presence of cladribine. (1×10^−8^ M to 5×10^−7^ M) for 72 h. Then cells were washed three times and transferred into cladribine-free medium. Cells were restimulated with anti-CD3/anti-CD28 antibodies for 48 h at days 9, 16, 23, 30, 44 and 58; supernatants were collected and cytokine concentrations were determined. Data are depicted as box plot diagrams. Whiskers represent maximum and minimum values. The IL-4/IFN-γ ratio was defined as the ratio of IL-4 (pg/ml) to IFN-γ (pg/ml). *: p<0.05; **: p<0.01.

**Fig 5:**
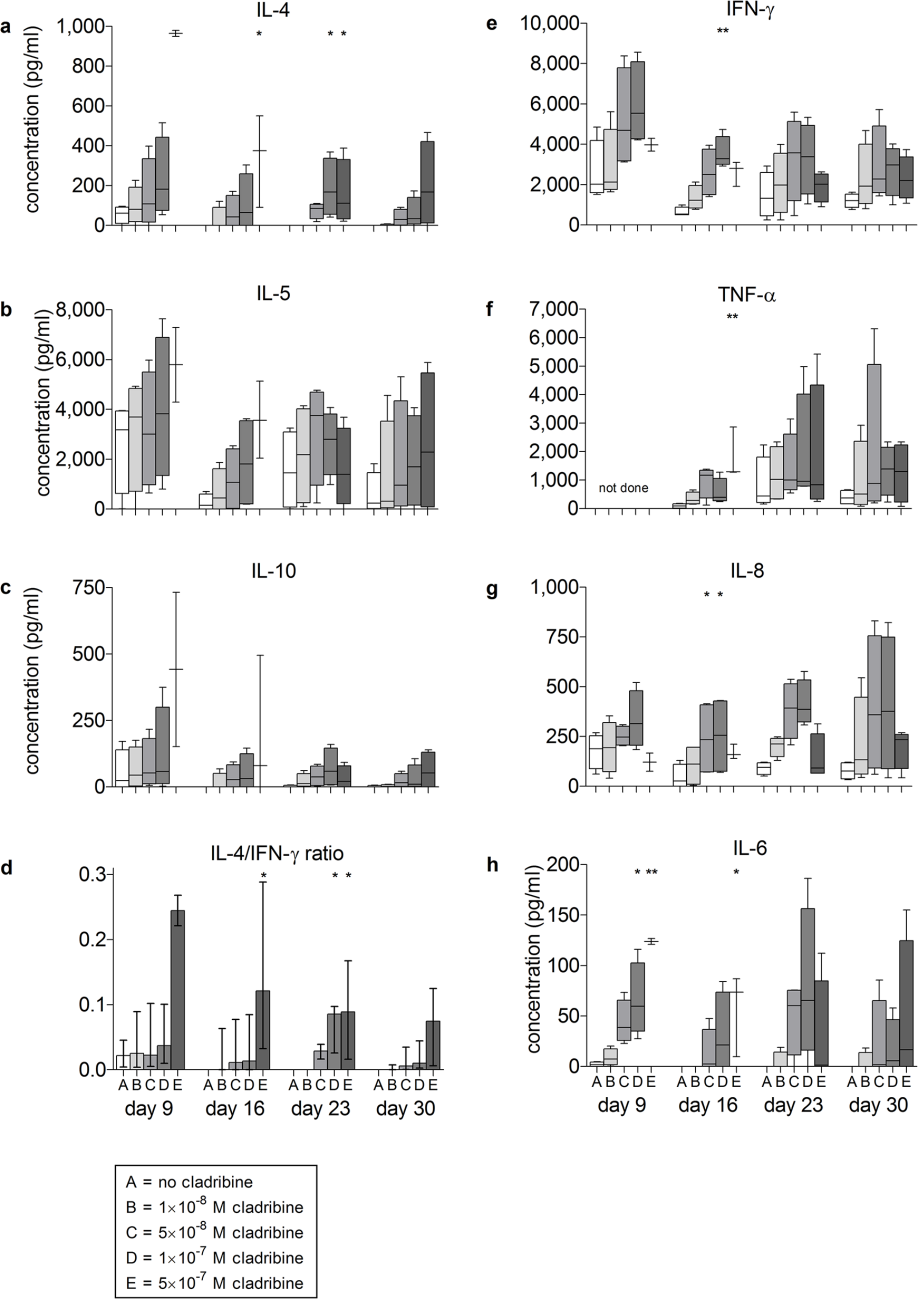
*In vitro* effects of initial cladribine treatment on cytokine secretion of PBMCs restimulated at Days 9, 16, 23 and 30 after transfer into cladribine-free medium. **PBMCs from healthy blood donors (n = 4) were incubated in the absence or presence of cladribine**. (1×10^−8^ M to 5×10^−7^ M) for 72 h. Then cells were washed three times and transferred into cladribine-free medium. Cells were restimulated with PHA for 48 h at days 9, 16, 23 and 30; supernatants were collected and cytokine were determined. Data are depicted as box plot diagrams. Whiskers represent maximum and minimum values. The IL-4/IFN-γ ratio was defined as the ratio of IL-4 (pg/ml) to IFN-γ (pg/ml). *: p<0.05; **: p<0.01.

To characterize the surviving cells in more detail, we measured the inducibility of prototypical pro- and anti-inflammatory cytokines (Figs 4 and 5). The release of anti-inflammatory cytokines by PBMCs in response to stimulation with anti-CD3/anti-CD28 antibodies was influenced by initial cladribine treatment in a concentration-dependent manner (Fig 4). Initial exposure to 5×10^-7^M cladribine had the strongest effects on cytokine release at all investigated time points. There was a statistically significant increase in the cytokine concentration for IL-4 and IL-5 at Day 9 (IL-4 p<0.01 and IL-5 p<0.01), Day 44 (IL-4 p<0.01) and Day 58 (IL-4 p < 0.05); similar trends were observed for IL-10. In contrast, no significant changes were observed regarding IFN-γ, tumor necrosis factor-alpha (TNF-α), IL-8 or IL-6. We also measured the secretion of IL-17A, IL-23 and nerve growth factor beta (NGF-β) at Days 9 and 30. No significant changes were observed (data not shown). To determine the overall effect of this enhanced cytokine production, the IL-4/IFN-γ ratio was determined. This analysis confirmed a shift towards an anti-inflammatory cytokine milieu (Day 9, p < 0.01; Day 44, p < 0.01; Day 58, p < 0.05) (Fig 4).

In an additional series of experiments, we stimulated the immune cells with the mitogen PHA instead of anti-CD3/anti-CD28 antibodies. The increase of TH2-type cytokines in cladribine-treated cells was reproduced under these conditions, demonstrating the robustness of this phenomenon (Fig 5).

## Discussion

This study is the first to address the question of whether cladribine exerts long-term immunomodulatory effects on the immune cells that survive the initial treatment, in addition to its demonstrated cytotoxicity. The concentration range of cladribine used for our *in vitro* study was chosen to reflect concentrations that occur in *in vivo* situations. Patients in the phase III clinical trial of cladribine for the treatment of MS patients received one or two 10 mg cladribine tablets once daily for four or five consecutive days. No plasma concentrations have been reported from this trial. Data are available from early reports by Liliemark et al. who detected peak plasma levels exceeding 100x10^-9^ M following the oral administration of 0.28 mg/kg cladribine [22]. More recently Munafo et al. presented plasma concentrations of 7x10^-8^ M after a single tablet of 10 mg oral cladribine. [23].

To explore whether the alteration of cytokine patterns persisted beyond the initial cytotoxic effects of cladribine, human PBMCs were cultured for up to 2 months after an initial 72h exposure to cladribine. Removal of the compound from the culture enabled the experimental separation of sustained immune modulation from the acutely toxic effects of cladribine.

The short-term treatment with cladribine resulted in a dose-dependent decrease of proliferation and a dose- and time-dependent increase of apoptosis. However, these acute effects did not persist in surviving cells: when the drug was removed from the cell culture after the initial 72h, cladribine did not inhibit the proliferation of PBMCs up to 58 days thereafter. Assessing the cytokine levels enabled us to show that initial cladribine treatment results in a sustained alteration of cytokine release in the surviving cells. Treated cells predominantly released anti-inflammatory cytokines in comparison to untreated cells, which was reflected by an increased IL-4/IFN-γ ratio. Cladribine did not change the secretion of NGF-β.

In earlier *in vivo* studies reductions of chemokines and soluble adhesion molecules were observed after the subcutaneous administration of cladribine [20,21]. Furthermore, Laugel et al. reported decreases in the production of IL-2, IL-10, IFN-γ and TNF-α in T cells that were treated with cladribine *in vitro* [24]. However, in these studies readouts were performed in the presence of cladribine; therefore, direct cytotoxic drug effects likely account for the reduced cytokine production.

Kraus et al. recently investigated the effects of cladribine on murine and human dendritic cells *in vitro*. T cells stimulated by murine dendritic cells that had been pre-treated with cladribine produced significantly less IFN-γ and TNF-α and significantly more IL-10. IL-4 production remained unchanged [25]. Together with our observation that cladribine-exposed human T cells produced more IL-4 but did not alter IL-10 production, these data suggest that cladribine exerts its immunomodulatory effects both by directly influencing T cell function and by altering the co-stimulatory function of dendritic cells.

We are aware that an *in vitro* study only partly reflects the *in vivo* treatment situation. The effect of cladribine on tissue resident cells and the effects that may occur through repopulation from bone marrow and lymphoid organs are not modelled in our *in vitro* system. Also, since the cells were obtained from healthy donors they may show a response pattern that differs from cells derived from patients with an ongoing autoimmune disease. Since marketing authorisation for cladribine was not granted in the US and Europe for patients with RRMS the manufacturer withdrew the drug from those markets where cladribine had been introduced. Therefore complementing this study with *ex vivo* data derived from treated MS patients is not feasible. The statistical approach chosen, was adapted to the exploratory character of the study. Statistical results were not corrected for multiple testing for each cytokine investigated. Therefore, additional studies are needed to confirm our findings. Also, the strongest effects reaching statistical significance were observed only at the highest concentration of cladribine. However, we believe that these data are biologically relevant since these findings were consistently observed using two different stimuli. Furthermore the graphical representation demonstrates a dose dependent induction of cytokines.

Despite these limitations our study is the first to apply an assay that allows the investigation of immunomodulatory effects of cladribine on surviving PBMCs that cannot be explained by direct cytotoxicity. Our data reveal that cells which survive cladribine treatment exhibit an anti-inflammatory phenotype with respect to their cytokine patterns. These findings are in line with the concept that the induction of an anti-inflammatory cytokine pattern contributes to the mechanism of action of immunosuppressive drugs in relapsing remitting MS [16]. Since enhanced induction of IL-4 is a common feature of cyclophosphamide, mitoxantrone and cladribine, it is tempting to speculate that IL-4 induction could be used as a lead marker to select additional immunosuppressive drugs for clinical testing.

In summary, we were able to demonstrate that an initial treatment with cladribine induces a sustained alteration in the cytokine profile of surviving PBMCs *in vitro*. This cannot be explained by persistent toxic effect after the removal of cladribine because proliferation remained unimpaired and cladribine administration resulted in an induction of IL-4 and IL-5. We conclude that the induction of anti-inflammatory cytokines may contribute to the beneficial effect of cladribine in RRMS patients.

## Supporting Information

S1 Dataset Excel file containing all source data. (XLSX)
